# A Study on the Ability of Nanomaterials to Adsorb NO and SO_2_ from Combustion Gases and the Effectiveness of Their Separation

**DOI:** 10.3390/nano14100816

**Published:** 2024-05-07

**Authors:** Marius Constantinescu, Felicia Bucura, Antoaneta Roman, Oana Romina Botoran, Roxana-Elena Ionete, Stefan Ionut Spiridon, Eusebiu Ilarian Ionete, Anca Maria Zaharioiu, Florian Marin, Silviu-Laurentiu Badea, Violeta-Carolina Niculescu

**Affiliations:** 1 National Research and Development Institute for Cryogenic and Isotopic Technologies—ICSI Ramnicu Valcea, 4th Uzinei Street, 240050 Ramnicu Valcea, Romania; marius.constantinescu@icsi.ro (M.C.);; 2 Faculty of Agricultural Sciences, Food Industry and Environmental Protection, “Lucian Blaga” University of Sibiu, 7–9 I. Ratiu Str., 550012 Sibiu, Romania

**Keywords:** adsorption, forecast, nanomaterials, concentration growth rate

## Abstract

Climate neutrality for the year 2050 is the goal assumed at the level of the EU_27+UK_. As Romania is no exception, it has assumed the gradual mitigation of pollution generated by the energy sector, and by 2030, according to ‘Fit for 55’, the share of energy from renewable sources must reach 42.5% from total energy consumption. For the rest of the energy produced from traditional sources, natural gas and/or coal, modern technologies will be used to retain the gaseous noxes. Even if they are not greenhouse gases, NO and SO_2_, generated from fossil fuel combustion, cause negative effects on the environment and biodiversity. The adsorption capacity of different materials, three nanomaterials developed in-house and three commercial adsorbents, both for NO and SO_2_, was tackled through gas chromatography, elemental analysis, and Fourier-transform infrared spectroscopy. Fe-BTC has proven to be an excellent material for separation efficiency and adsorption capacity under studied conditions, and is shown to be versatile both in the case of NO (80.00 cm^3^/g) and SO_2_ (63.07 cm^3^/g). All the developed nanomaterials generated superior results in comparison to the commercial adsorbents. The increase in pressure enhanced the performance of the absorption process, while temperature showed an opposite influence, by blocking the active centers on the surface.

## 1. Introduction

Long-term exposure to air pollution affects the health of humans, plants, and animals, contributing significantly to ecosystem degradation and is one of the causes of premature mortality of people [1,2]. Hazardous atmospheric compounds impact the sustainability of the environment and public health, and are collectively referred to as “atmospheric air pollution”. Anthropogenic and industrial activities, notably the combustion of fossil fuels, generate substantial quantities of greenhouse gases (GHGs) and other air pollutants such as dust, PM_2.5_, CO, NO_x_, and SO_2_. Human development came along with urbanization, which contributed enormously to socio-economic progress, but caused damage to the global environment [3], due to the increase in energy consumption, the use of fossil fuels, and significant industrial emissions [4]. These primary pollutants can undergo through chemical reactions in the atmosphere, leading to the formation of secondary pollutants [5]. 

NO and SO_2_ gases play an important role in the formation of smog, producing the brown haze often seen in urban areas, especially during summer months. Through exposure to UV rays in sunlight and heat, NO molecules interact with volatile organic compounds (VOCs) and form the tropospheric ozone (O_3_) level, a major pollutant that affects air quality and public health [5].

Acid rain, another environmental concern, is a phenomenon that results from the interaction of NO and SO_2_ with atmospheric precipitation, leading to the formation of nitric and sulfuric acids. This occurrence is extremely dangerous for human health as well as having a negative influence on the ecosystem. Consequently, removing these pollutants from flue gases becomes crucial for environmental protection [6], and can be achieved by several methods including desulphurization, limestone–gypsum technology, alcohol-amine technology, and washing with alkalis [7,8]. However, the resulting mixtures often contain traces of sulphureous and nitrous gases (at ppm levels), which should be further treated through adsorption technology [9].

Metal–organic frameworks (MOFs) are porous adsorbents composed of inorganic metal-oxide secondary building units connected by polydentate organic linkers. This cross-linked network typically provides a high surface area and porosity, and has found potential applications in the separation, detection, and removal of toxic chemicals. MOFs offer significant advantages over other traditional porous materials such as activated carbon and zeolites due to tunable pore structures and chemical functionality [10,11], but also low operating pressure and cost.

Being regarded as having a high catalytic activity among the MOFs, Fe-BTC (BTC: 1,3,5-benzenetricarboxylate) [12] has attracted attention due to its enhanced interaction strength with guest molecules and its broad catalytic capacity.

Recently, UiO-66 (Zirconium 1,4-dicarboxybenzene metal–organic framework), a distinctive MOF, has garnered interest due to its high working capacity, good selectivity, moisture stability, thermostability, and affordability [13,14,15,16]. The crystalline structure of UiO-66 resists moisture and acidic conditions [17,18], has similar physicochemical properties to the core framework, and exhibits better gas separation performance [19,20].

Similarly, Cu-Zn-MCM-41 is a material with a linear uniform nanostructure and porous texture, forming an ordered arrangement of hexagonal channels with homogenous pore sizes [21]. Its uniform adsorption of NO and SO_2_ shows that Cu and Zn loading does not compromise the mesopore structure [22].

The assumed objective at the level of the EU_27+UK_ is climate neutrality by 2050. Romania has adopted a progressive approach to mitigate the pollution resulting from the energy sector, and by 2030, 42.5% of the country’s total energy consumption must come from renewable sources, according to ‘Fit for 55’ [23]. For the remaining energy generated from conventional sources, such as coal and/or natural gas, advanced technologies will be employed to remove the combustion gaseous pollutants.

This study aims to explore solutions to reduce emissions of SO_2_ and NO, resulting from the burning of fossil fuels in boilers at thermal or electrical power plants. To this end, the ICSI Ramnicu Valcea laboratory initiated sampling campaigns for flue gases generated during lignite combustion, from the CET Govora plant in Valcea County. The specific analytical investigations of the samples revealed concentrations of noxes around 200 ppmv for NO and 100 ppmv for SO_2_. These findings formed the basis for creating binary mixtures, which were then used to evaluate the adsorption capacities of the different materials targeted by the study. A pivotal component of this investigation is the comparative analysis of the adsorption behaviors of different nanomaterials developed by ICSI Ramnicu Valcea—including Fe-BTC, UiO-66, and Cu-Zn-MCM-41—compared to commercial materials such as Activated Carbon (AC), ZSM-5, and SiO_2_.

## 2. Materials and Methods

**Figure 1:**
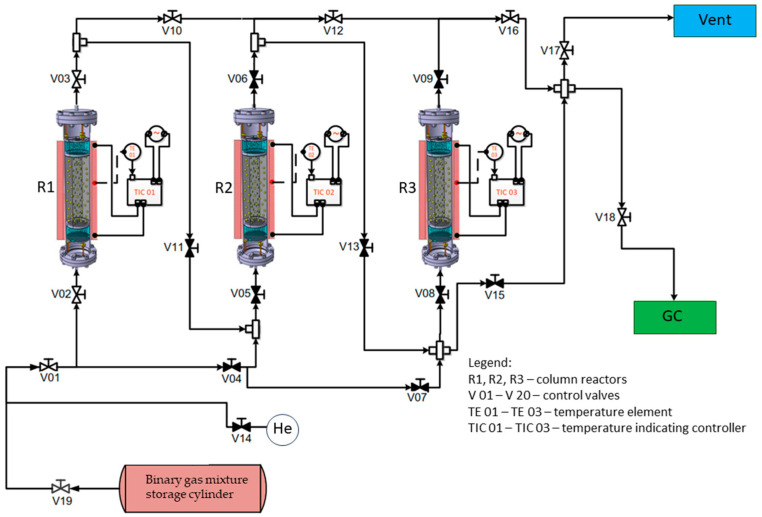
Three-column reactor system experimental setup.

Experimental testing of adsorption capacity, *a* (cm^3^/g), was conducted in a three-column reactor system with versatile functionality for independent or parallel use [24], Figure 1. The study focused on the comparative analysis of the different behaviors of the tested nanomaterials, developed by ICSI (Ramnicu Valcea, Romania) (Fe-BTC, UiO-66, Cu-Zn-MCM-41), and commercial materials (Activated Carbon-AC, ZMS-5 and SiO_2_, all three being purchased from SC UTCHIM SRL, Ramnicu Valcea, Romania). The Fe-BTC, UiO-66, and Cu-Zn-MCM-41 nanomaterials were previously synthesized and characterized by the authors at ICSI (Ramnicu Valcea, Romania) facility [24], while the commercial materials such as Activated Carbon (AC), ZSM-5, and SiO_2_ were purchased from SC UTCHIM SRL in Ramnicu Valcea, Romania.

Prior to every testing experiment, leak tests were performed using the high-purity inert gas, helium, in order to confirm the system’s tightness. To ensure the comparability of data, the experimental technical conditions for the various adsorption tests were standardized as follows: (i) maximum flow rate (Q_max_) of 100 mL/min; (ii) nominal pressure (P_nominal_) set at 2 bar and 6 bar; (iii) gas velocity of 3 m/s; (iv) column-filling layer height of approximately~135 mm. These static parameters were completed by variable ones: pressure (P) at 1 bar and 5 bar, temperature (T) at 313.15 K and 373.15 K, adsorbent material mass, and the initial concentration of the tested gases. All the developed experiments were conducted over a time period of 35 min. The PG 250 (Horiba, Japan) analyzer was used in this study for qualitative and quantitative NO investigation, engaging the chemiluminescence analysis principle. The evolution of the SO_2_ concentration during the tests was monitored using the GC Nexis 2030 (Shimadzu, Kyoto, Japan), the method presenting the following technical characteristics: FPD detector; T_detector_: 323.15 K; column type—GC GASPRO; T_column_: 323.15 K; T_injector_: 383.15 K; carrier: He (5.0).

The adsorbent materials were investigated both prior to the adsorption capacity testing, but also at the end of the processes, in order to determine the particular compositional and structural modifications that occur, employing two different techniques. The elemental analysis, using EA2000 analyzer (Thermo Fisher Scientific, Swindon, UK), represents a hybrid investigation method, combining the combustion of the adsorbent sample with gas chromatography, consisting of the separation of the components of the gaseous mixture on a packed chromatographic column and then their qualitative and quantitative determination of chemical elements (N and S) with significant environmental impacts. For the calibration curve a reference material was used, BBOT (Thermo Fisher Scientific, Regensburg, Germany), an amino acid with 7.42 wt% S and 6.48 wt% N content. Since the elemental content variation was small, an additional validation of the results was performed with another reference material, Sulfanilamide (Thermo Fisher Scientific, Regensburg, Germany), with a content of 18.63 wt% of S and 16.23 wt% N.

The second technique, FT-IR analysis, used a CARY 630 spectrometer (Agilent Technologies, Inc., Santa Clara, CA, USA). Prior to analysis, the original adsorbent materials were ground in an agate mortar and dried at 80 °C under vacuum. The spectra were recorded using the Diamond-ATR (Attenuated Total Reflectance) module between 4000 and 400 cm^−1^, at a resolution of 8 cm^−1^, and 32 scans, with a threshold of 0.002. The spectra were elucidated using MicroLab Expert v.1.0.0 Software (Agilent Technologies, Inc., Santa Clara, CA, USA). For the calibration curve a reference material was used, BBOT (Thermo Fisher Scientific, Regensburg, Germany), an amino acid with 7.42 wt% S and 6.48 wt% N content. Since the elemental content variation was small, an additional validation of the results was performed with another reference material, Sulfanilamide (Thermo Fisher Scientific, Regensburg, Germany), with a content of 18.63 wt% of S and 16.23 wt% N.

The data obtained from the previously described investigations were used for the validation of the testing system, nanomaterial, and experimental setup, and or the calculation of the specific physical parameters: (i) a—adsorption capacity, cm^3^/g; (ii) η—separation efficiency, %; and (iii) R = degree of recovery, ppmv [24].

For testing the adsorption capacity of nanomaterials and commercial adsorbents, in accordance with the experimental setup, two binary gas mixtures were developed, 150 ppmv NO balanced N_2_ and 75 ppmv SO_2_ balanced N_2._ The method of preparing binary mixtures was that of partial pressures [25], using dried high-purity gases (Linde, Pullach, Germany), and executed in cylinders with 10 L volume made of stainless steel, a material that does not allow the diffusion of gases.

## 3. Results

### 3.1. Specific Characteristics of the In-House Developed Nanomaterials

The used nanomaterials (Fe-BTC, UiO-66, and Cu-Zn-MCM-41) were previously synthesized and fully characterized [24]. Briefly, some morpho-structural characteristics are provided below, to better understand their adsorption mechanisms. The specific surface areas of the samples were 360 m^2^/g (Fe-BTC), 811 m^2^/g (UiO-66) and 607 m^2^/g (Cu-Zn-MCM-41), according to the Brunauer–Emmett–Teller (BET) methods [24]. The pore size distribution showed in the case of Fe-BTC micropores with an average diameter of around 1.9 nm, as well as mesopores with an average diameter of abound 2.4 nm, the sample having a porous structure at the boundary between micro- and mesopores [24]. The pore size distribution of UiO-66 presented micropores with an average diameter of 1.5–1.7 nm, as well as mesopores (diameter of 2.9 nm) [24]. In the case of Cu-Zn-MCM-41, the pore size distribution highlighted mesopores of 3.5 nm diameter [24]. Regarding the morphology, Fe-BTC had a homogeneous, dense structure, with granular-shaped particles, the average particle size being 200 mm [24]. UiO-66 had a homogeneous dense structure, consisting of nano-sized crystals of octahedral shape with a narrow size distribution between 150 and 250 nm [24]. The bimetallic nanomaterials Cu-Zn-MCM-41 exhibited spherical and partially elongated particles below 100 nm, with the ordered morphology typical of MCM-41-type mesoporous silica being evidenced [24].

### 3.2. Study of the Adsorption Capacity of Nanomaterials for NO

#### 3.2.1. Adsorption Mechanism

**Figure 2:**
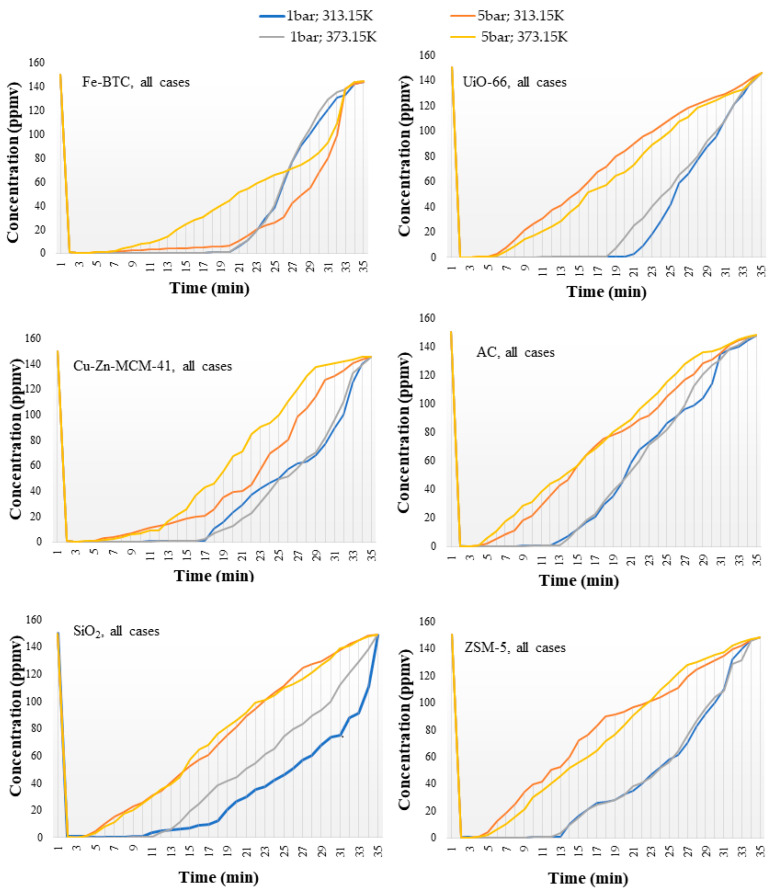
Evolutional profile of NO concentration along the adsorption process in the case of different nanomaterials and commercial adsorbents.

The investigation of the adsorption capacity, along with other physical parameters, was undertaken starting from the chemiluminescence method analysis of the binary gas mixture, which monitored the evolution of the NO concentration for each tested case, as showed in Figure 2. The evolution of the NO concentration was followed during the 35 min of the process, divided based on the trend described in three distinct intervals: (I) from the initial concentration (C_i_), 150 ppmv to the corresponding breakthrough time, <1 ppmv; (II) the 1–100 ppmv concentration range and (III) the 100 ppmv—final concentration (C_f_). The goal of selecting this interval-based data analysis methodology was to facilitate the observation of the adsorptive behavior of the nanomaterials.

The first interval is distinguished by a steep decrease in the initial concentration marked by the adsorption process, regardless of the nanomaterial type. The analysis of the data presented in Table 1 indicates a strong influence of the pressure variation on the period of this interval, the breakthrough time at 5 bars being considerably lower than at 1 bar, for all tested cases. The highest breakthrough time, ~19 min, was recorded in the case of Fe-BTC, materials with high strength, with unsaturated metal sites inside the pore walls, suitable for binding small molecules such as NO [26]. Adsorption site for NO was mainly governed by the Fe spread inside the Fe-BTC molecule [27], followed by the physical driving forces, pressure, and temperature [28]. The molecules of NO were preferentially adsorbed on unsaturated sites for layers below NO/Fe = 1:1 ratio.

The zirconium-based MOF family is known as a high-stability MOF due to its coordination between the metal node and the organic linker, which prevents the metal node from chemical attack [29]. Among MOFs, UiO-66 has been the most investigated in adsorption applications, showing remarkable performances [30,31]. The interactions between UiO-66 and NO molecules, from the first interval, was encouraged by the presence of Van der Waals-type bonds, π-π stacking, and hydrogen bonds [32], which resulted in almost the same breakthrough time as in the case of Fe-BTC. Also, the NO molecular size, 3.17 Å, allowed maximum contact with the adsorbent, UiO-66, making the process more efficient. The breakthrough time was increased under the electrostatic effect given by the metal frameworks, which have governed the electrostatic adsorption of the NO molecule.

Cu-Zn-MCM-41 has an ordered arrangement of hexagonal channels with uniform pore size and high specific surface, a mesoporous structure that enhanced the mass transfer process, increasing the breakthrough time. The adsorption kinetics were fast, especially in the first 3–16 min, Table 1. The effect of pH demonstrated that a weak acidic condition favored the adsorption process. NO adsorption was achieved at pH < 6, where Cu and Zn, well dispersed in the silicon pore walls, contributed decisively to a strong NO adsorption [33]. The results obtained by this study showed that the presence of Cu and Zn could increase the adsorption capacity, facilitating the oxidation of NO. Therefore, it could be inferred that chemical adsorption probably had a better removal effect than physical adsorption [34]. Upon introduction of transition metals, Cu and Zn, into the MCM-41 structure, the protons of the Brønsted acid sites are replaced by Cu (II) and Zn (II) cations, increasing the number of Lewis acid sites. Interestingly, Cu-Zn-MCM-41 exhibits a large total amount of Lewis and Brønsted acid sites, also responsible for NO adsorption, on the transition metal sites. This indicates that Zn acts as a promoter, affecting the nature of Cu in the nanomaterial, and providing additional sites for NO adsorption [35].

Activated carbon (AC), with an average micropore size of about 7 Å, shows a varied porous textures and specific surface, beneficial for NO removal [36]. The NO adsorption was dependent on the adsorbent material oxygen content, with AC acting both as (i) a catalyst for NO oxidation, positively correlated with the narrow micropores, but independent of the specific surface and (ii) as an adsorbent for NO. The rapid oxidation of the AC surface and the chemisorption of NO are believed to be facilitate by the decomposition of NO_2_, which is formed by the oxidation of NO. This adsorption process involves the formation of chemical bonds between the adsorbed substance and the AC [37]. The effectiveness of the adsorption process by the AC in the tests performed with the use of the column-reactor system was also impacted by the chemical reaction between NO and certain functional groups, such as carbonyl and carboxyl, which are part of the chemisorption process [38]. Physisorption, a weak intermolecular attraction that occurs below the adsorbate’s critical temperature, facilitated by the Van der Waals force, completes the process of NO adsorption over the commercial activated carbon. The exothermic nature of the reactions made the process most feasible at ambient temperatures [39,40].

Under ambient conditions, SiO_2_ did not react with NO, but reactivity was promoted with the increase in pressure. This relevant interaction between the SiO_2_ nanostructure and high pressure served for the optimization of NO transfer in small reactive pores, as well as the stabilization of the absorbent’s pore structure [41].

Zeolitic materials have gained significant attention as passive absorbers of NO [42]. ZSM-5 is a member of the MFI-zeolite family, with a high Si/Al ratio of 10:100. It features three-dimensional channels, straight channels with 0.54 nm × 0.56 nm pore openings and sinusoidal channels with a pore opening of 0.51 nm × 0.55 nm. ZSM-5 received great attention from researchers due to its thermal stability, high specific surface and strong acidity. The acid sites allowed the zeolite to be used as a catalyst in selective adsorption processes. The size of the zeolite crystal is much larger than the micropore size and this results in a low diffusion rate, leading to a small mass transfer. Since physical adsorption is always exothermic, the adsorption capacity increased with decreasing temperature, and the adsorbent sample approached saturation with pressure increase. The physical adsorption of NO in ZSM-5 is characterized by the occupation of cationic positions in the zeolite [43]. It has been shown that the primary adsorption sites of NO are the hydroxyl frameworks of Si(OH)Al, which are strongly protic bridges, corresponding to the first interval [44]. The concentration growth rate, attributed to the decrease in the adsorption capacity during the tested period, represented another analyzed parameter in an attempt to comparatively evaluate the studied nanomaterials. Its calculation considered 1 min segments of the evaluated intervals. For the first interval, the growth rate calculation considered the range between the first value recorded by the equipment after the initial value and the concentration corresponding to the specific breakthrough time. The values of the growth rate ranged between 0.28 ppmv and 0.55 ppmv, with a negligible variation from one tested temperature to another, regardless of the studied case. The pressure showed a higher impact, with the most homogenous data series presented in the case of Cu-Zn-MCM-41 and the most heterogenous for AC.

The second interval showed a progressive deceleration of the adsorption capacity, described by an upward slope of the NO concentration, Figure 2. This interval completes the timeline of the previous interval, its duration being positively linked to the pressure. At this stage of adsorption, once the primary adsorption sites have been filled other weaker acidic places (chemisorption) and less-accessible caverns (for physisorption) began to adsorb NO [44]. For the second interval, the growth rate, with values located in the range of 23 ppmv–34 ppmv, shows a greater uniformity, suggesting a lower dependence degree of the process in relation to temperature and pressure variations.

The third interval is characterized by a longer adsorption duration under higher pressure, with the exception of Fe-BTC. A more detailed analysis of the case attributed this atypical behavior to the extensive duration of the second interval, in which NO was significantly absorbed within the specific caverns, due to an optimal NO:Fe ratio of 1:1, up to the point at which these spaces reached saturation [45]. The concentration growth rate varied from 2 ppmv to 26 ppmv, its dependence on temperature being higher than in the cases of the previous intervals. This phenomenon is due to the fact that the active adsorption centers are thermally deactivated by thermal agitation, where elevated temperatures represent a negative impact on the selective aggregation, also leading to a decrease in their density [46].

The separation efficiency, η (%), serves as a tool used to evaluate the performance of various cases, quantifying the ratio of NO separated in the column-reactor system to that separated by a specific nanomaterial. The achieved separation efficiency, ranging from 96.06% to 99.41%, signify highly efficient separation processes across all tested scenarios, as presented in Table 1. Comparative analyses of different adsorbent materials, in general, have shown an inverse proportionality; for the high separation-efficiency values, the analogous adsorption capacities decrease, respectively [47], a similarity also found in the present study.

According to the principle of circularity, the adsorbed gas can be used in future experiments/processes, but only after recovery (R, %) by depressurization, exhaustion, and suction for~35 min at 10^−2^ bars [48]. The average values of the recovery are higher in the initial processes carried out at pressures of 1 bar, R~70%, Table 1. The most homogeneous recovery processes of the NO adsorbed, regardless of the parameters used in the tests, proved to be those under Fe-BTC, CV < 10%, and the most heterogeneous those under SiO_2_, CV > 20%.

#### 3.2.2. Validation of Nitric Oxide Absorption Capacity through Compositional and Structural Characterization of Nanomaterials

The present study reveals a combined theoretical and experimental investigation of the different nanomaterials, in order to evaluate their adsorption capacity for NO. The method for determining N involved rapid combustion of the sample. Previously, the collected sample was homogenized with a ball mill, weighed in a tin container and introduced into the combustion reactor through the autosampler. After combustion, the resulting gases were carried by a flow of pure helium (5.0) over electrolytic copper, copper oxide, and a GC column (SM 5A) and finally detected by a thermal conductivity detector (TCD) [49]. All data were obtained with good reproducibility, RSD < 0.05%, and there was no influence on the outcomes when a different nanomaterial was tested.

The elemental analysis results for tested adsorbent materials with varying initial nitrogen contents are listed in Table 2, indicating, for UiO-66, SiO_2_ and ZSM-5, a content below the quantification limit. The values of N evolution in the nanomaterials follow the same pattern as in the case of those revealed in Table 1, where high pressure was the defining parameter in the adsorption process, the exception being noted only in the case of Fe-BTC, with an inverse correlation. In absolute numbers, Fe-BTC (5 bar, 313.15 K) shows the highest N-gained content of ~3 wt%, positively correlated with the highest adsorption capacity observed across all cases, at 80 g/cm^3^. In contrast, Cu-Zn-MCM-41 (1 bar, 373.15 K), developed the lowest N-gained content of ~0.01 wt%, respectively 46 g/cm^3^. Although being comparatively lower in relation to those of the developed nanomaterials, the performances of the commercial adsorbents are notable, and their effectiveness, in descending order, was the following: AC (5 bar, 313.15 K)~0.3 wt% > ZSM-5 (5 bar, 313.15 K)~0.10 wt% > SiO_2_ (5 bar, 373.15 K)~0.05 wt%.

**Figure 3:**
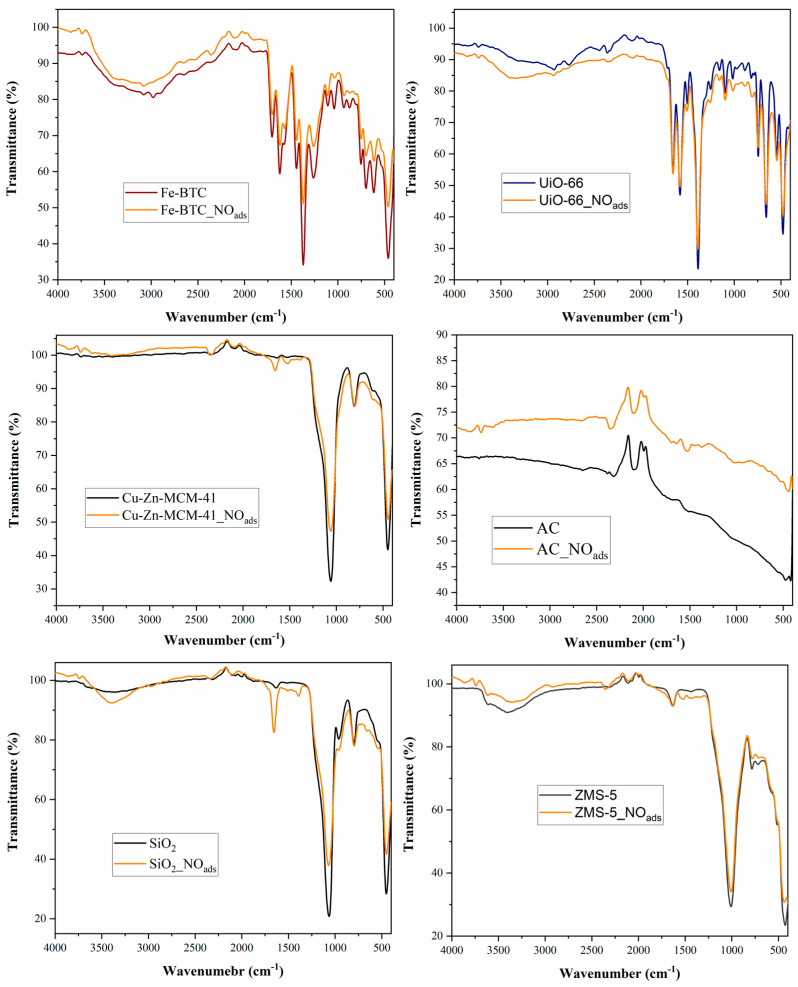
FT-IR spectra for the different nanomaterials and commercial adsorbents before and after the NO adsorption at 5 bars.

FT-IR spectroscopy confirmed the results of the elemental analysis, as well as those of the chemiluminescent investigation, shown in Figure 3, observing the attenuation of the peaks specific to the functional groups in the catalyst structure, as well as the appearance of two new peaks, around the values of 1600 cm^−1^ and 2400 cm^−1^, confirming the NO adsorption [50]. In the case of Fe-BTC and UiO-66, the peak from 1600 to 1650 cm^−1^ overlapped the nanomaterial’s characteristic ones. Although in the case of Fe-BTC, FT-IR analysis does not elucidate the NO adsorption, this nanomaterial adsorbed the highest quantity among the tested ones, around 2.5%. The materials with the lowest adsorption capacity were Cu-Zn-MCM-41 and SiO_2_, proving that materials based on silica are not suitable to be used as NO adsorbents.

### 3.3. Study of the Adsorption Capacity of Nanomaterials for SO_2_

#### 3.3.1. Adsorption Mechanism

**Figure 4:**
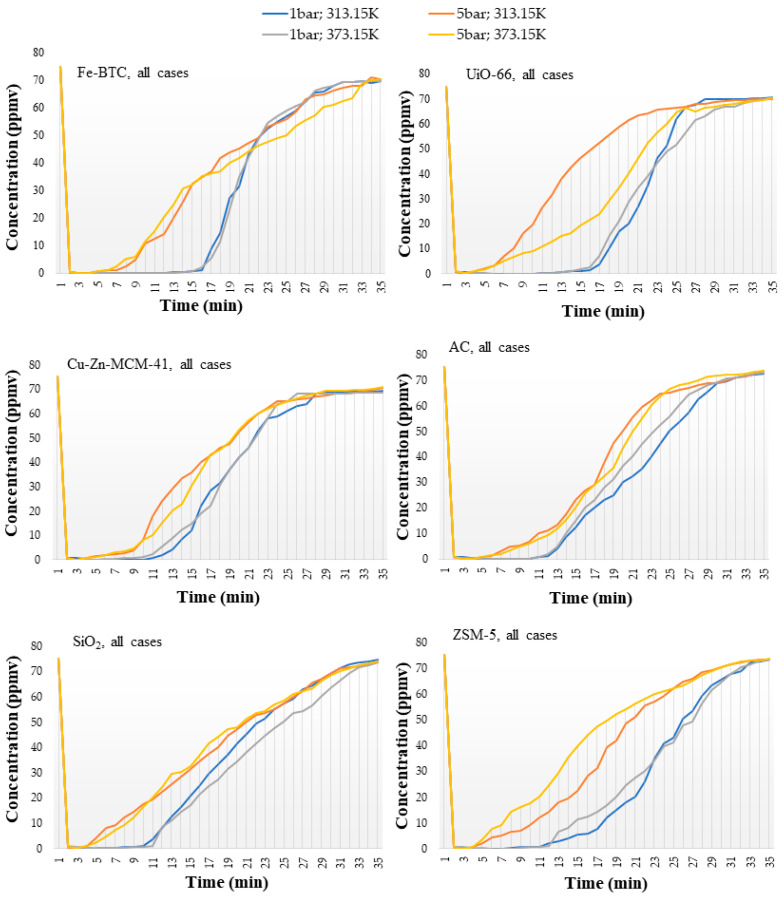
Evolutional profile of SO_2_ concentration during the adsorption process in the case of different nanomaterials and commercial adsorbents.

Sulfur dioxide generated by the burning of fossil fuel is a major pollutant, which can generate acid rain and photochemical smog, and many other unwanted environmental and health hazards [51]. Therefore, the capture or separation of SO_2_ is an important gas treatment process in industry. The investigation technique was similar to the one used for the NO adsorption study, in terms of process time, 35 min, and interval division. As the initial concentration was 75 ppmv, the borders of these intervals were different, more precisely (I) 75 ppmv to the corresponding breakthrough time, <1 ppmv; (II) 1–50 ppmv concentration range and (III) 50 ppmv—final concentration (C_f_). The gas chromatography method was used to track the evolution of the SO_2_ concentration during the testing, as shown in Figure 4.

For SO_2_ adsorption tests, the duration of the first interval was significantly influenced by pressure, with breakthrough time varying from 3 min to 15 min, Table 3. From this range, Fe-BTC generated the highest values for SO_2_ adsorption, as well as for NO. Studies of SO_2_ adsorption isotherms on Fe-BTC, similar to other MOF materials, showed that a high SO_2_ adsorption capacity was conditioned by a nanomaterial pore size greater than 4 Å. This conditionality is owed to the higher kinetic diameter of the SO_2_ molecule compared to NO, of 3.6 Å.

The amount of SO_2_ adsorbed in this pore size range at low pressures relates to the heat of adsorption, with Fe-BTC displaying a larger SO_2_ storage capacity at low pressure than most hydrotalcite-type materials [52]. Free volume and surface area are important features in evaluating any absorbent and storage potential of nanomaterials; in the case of Fe-BTC, their adsorption mechanism at moderate pressure was demonstrated, Table 3. This result suggests that Fe-BTC has potential as a catalyst of sulfur transfer in industry. The molecular mechanisms responsible for SO_2_ adsorption were represented by Van der Waals interactions, the driving forces that controlled adsorption in this system [53]. Binding sites, the open-metal sites within the framework, attract and adsorb SO_2_ [54].

With the advantage of high selectivity, moisture stability, thermostability, acid gas resistance, high adsorption capacity and low-cost renewability, UiO-66 is considered a promising material in adsorption and purification applications of various gases. The molecular simulations used to investigate the performance of adsorption and separation of SO_2_ from in-house binary gas mixtures indicate an adsorption performance similar to that of Fe-BTC. As a mechanism, the adsorption of isosteric heat at infinite dilution, complemented by the radial distribution function, revealed strong interactions between hydrophilic functional groups and SO_2_. The hydrophilicity effect and the strong polarity of the functional groups favored SO_2_ adsorption. The strongest guest–host interactions in smaller tetrahedral cages were observed to dictate the better filling of accessible sites within the cavities in the case of SO_2_. UiO-66 is a suitable candidate for SO_2_ separation, due to its superior selectivity and permeability [55].

Cu-Zn-MCM-41 is representing an efficient alternative to similar products in this niche, from a technical and economical point of view. The Cu^2+^ and Zn^2+^ ions within the material’s framework created active sites for adsorption, interacting with the SO_2_ molecules and leading to their immobilization [56]. The ordered mesoporous channels of MCM-41 provided a favorable environment for gas adsorption, with SO_2_ molecules being diffused into their pores, where they encountered metal ions [57].

The highest adsorption capacity among all tested commercial adsorbents was attributed to *AC,* a porous carbonaceous material, being facilitated by the pore structure, the specific surface and the functional groups of the adsorbent [58]. The pore size and distribution showed a greater impact on SO_2_ adsorption capacity than the pore volume [59]. The adsorption mechanism involved (i) the physisorption, under working pressure and (ii) chemisorption, in which the adsorbed SO_2_ molecules were catalytically oxidized into sulfuric acid due to the intramolecular presence of oxygen and water vapor [60], and being safely sequestered in the pores of the AC. Physisorption involved repeated adsorbate–adsorbent interactions, with the effect of condensing polar SO_2_ molecules, through van der Waals interactions with the π-electrons of the aromatic rings located on the AC surface, in the form of islands or clusters [61]. The SO_2_ adsorption was instantaneous in the case of AC, imprinting an upward trend of the free energy of the adsorption system and the degrees of freedom, leading to an increased enthalpy. SO_2_ adsorption was an exothermic process with a similar behavior to the NO absorption process, regardless of the types of adsorption force, and its efficiency decreasing with the increase in temperature [62].

For SiO_2_, the large volume of the pores, the specific surface, and the large functional mesoporous silica, which exhibits a framework texture of wormholes, made possible the physical sequestration of SO_2_ in the adsorbent-material functionalized structure [63,64]. This type of adsorption follows Henry’s law, being proportional to the internal surface area [65]. The adsorption was in accordance with the descriptions in the specialized literature, namely an interaction with the hydroxyl groups, a mechanism with two means of manifestation: (i) intermolecular, with the formation of a hydrogen bond between the functional group and the oxygen in SO_2_ and (ii) intramolecular, forming SO_2_ by two additional covalent O-S bonds [66].

The adsorption method in the case of ZSM-5 involved a similar mechanism to that of SiO_2_, with SO_2_ being adsorbed both through physisorption and chemisorption by hydrogen bonding to hydroxyl groups [67]. Physical adsorption was possible due to the ZSM-5 structure, featuring straight linked channels, in which the active centers adsorb one or more gas molecules per situ, the adsorption position being non-parallel [68].

The analysis of the concentration growth rate on the first interval shows a higher homogeneity in the case of commercial adsorbents, with a standard deviation of about 0.05 ppmv, compared to approx. 0.08 ppmv in the case of those prepared in-house. Similarly to the NO study, for SO_2_ adsorption the temperature had no enhancing impact on the concentration growth rate.

The second and third intervals mark the same trends in terms of growth rate, duration, and thermal and pressure sensitivity, as described for NO adsorption processes, indicating a comparable behavior of the adsorbent materials for both adsorbed gases.

The high potential of the tested adsorbent materials in separating SO_2_ from flue gases is also confirmed by the elevated separation efficiency values described in Table 3, ranging between 91.25% (for Cu-Zn-MCM-41, 1 bar, 373.15 K) and 99.81% (for SiO_2_, 1 bar, 313.15 K). The type of adsorbent material is the only factor that locates the ɳ_SO2_ in the lower or upper limits of this range, independent of the technical parameters, temperature, and pressure.

Medium values of the recovery rate place Fe-BTC at a slight advantage to the other nanomaterials (56.09% R_SO2_), in a tight domain, Table 3. For all adsorption tests, higher pressure diminished the recovery rate.

#### 3.3.2. Validation of Sulphur Dioxide Absorption Capacity through Compositional and Structural Characterization of Nanomaterials

The amount of sulfur in the tested samples with 75 ppmv SO_2_ was distributed both on the surface and in the pores/caverns of the nanomaterial; therefore, a homogenization process was necessary prior to the elemental investigation. Even if the performances were not recorded, as in the case of N testing, there are similarities regarding the Fe-BTC, 5 bar, 313.15 K case, Table 4, and the highest sequestration capacity at the end of the test of S ~ 1 wt%, positively correlated with the highest adsorption capacity of 60 g/cm^3^. In decreasing order, Cu-Zn-MCM-41, 5 bar, 313.15 K, gained~0.9 wt% S, linked with a_SO2_ of 43 g/cm^3^, followed by UiO-66, 5 bar, 313.15 K, with a S-gained~0.6 wt%, respectively, and an associated a_SO2_ of 52 g/cm^3^. The ranking of SO_2_ absorption capacity is also validated in the case of commercial adsorbents by cross-cutting with the gained S content, AC > ZSM-5 > SiO_2_, respectively.

**Figure 5:**
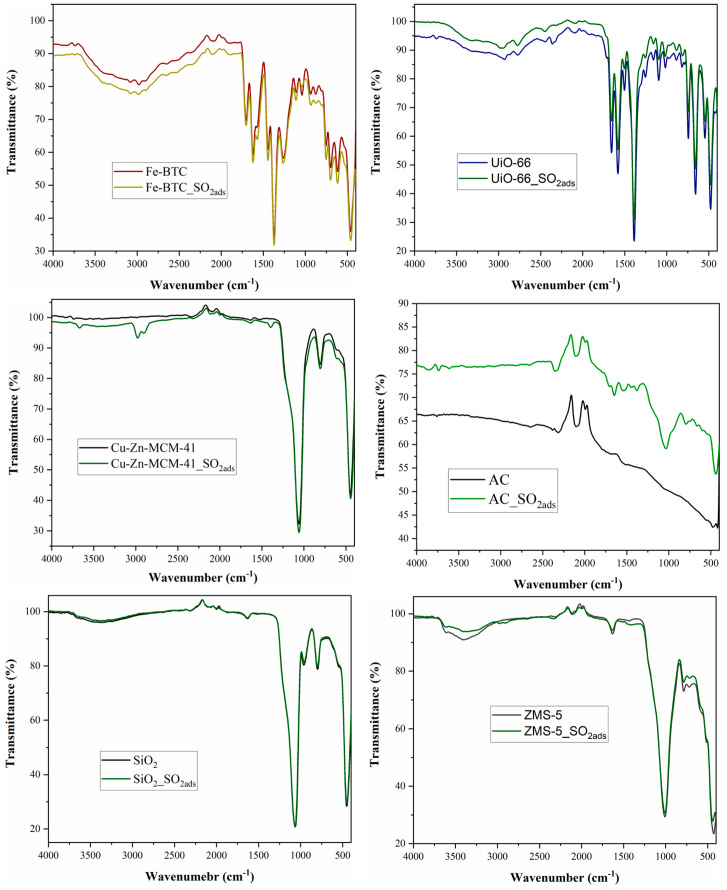
FT-IR spectra for the different nanomaterials and commercial adsorbents before and after SO_2_ adsorption at 5 bars.

Figure 5 presents the functional groups of the nanomaterials and commercial adsorbents before and after SO_2_ adsorption. FT-IR spectroscopy confirmed the results of the elemental analysis, as well as those of the gas chromatographic investigation, observing the attenuation of the peaks specific to the functional groups in the material structure and the appearance of new peaks specific to the newly formed bonds with the SO_2_ molecules. According to the literature, an increase in the number of hydroxyl groups facilitates the adsorption of sulfur dioxide, better observed in the case of Cu-Zn-MCM-41 by the appearance of a two-split peak at 2900–2970 cm^−1^ [69]. In the case of Fe-BTC and UiO-66, the specific peaks for the interaction were overlapped with the material’s specific peaks. In the case of SiO_2_, the quantity of adsorbed SO_2_ was very reduced, a fact confirmed by the elemental analysis. An interesting case can be observed for AC, which formed chemical bonds with the SO_2_ molecules, proven by the intense peak around 1030 cm^−1^ and weak ones from 1300 to 1600 cm^−1^. The peak from about 1030 cm^−1^ was assigned to S=O stretching frequency, indicating sulfate or sulfite formation on the AC [70]. The weaker peaks from the 1300–1600 cm^−1^ band were assigned to the C=O stretching mode in carbonyls, carboxylic acids and lactones, to C-O stretching and to O-H bending from phenols and carboxylic acids [58].

### 3.4. Forecast Approach

The accurate forecast of the studied nanomaterials’ adsorption capacities over NO and SO_2_ is critical for sustainable development and management perception. Also, the reduction in material and time costs for the performance of additional tests, represents another asset in approaching a precise forecast to generate new data on the experimental adsorption capacity. Sorting data to deliver important information using forecasts is a modern approach to assessing gaseous noxes, especially projections based on the research and development of nanomaterials [71]. The present study employed the mathematical technique of linear interpolation and extrapolation, by filling gaps between experimental data [72], to forecast the adsorption capacity of in-house developed nanomaterials and commercial adsorbents. The forecast approach between experimental data points took into account the same variation range: for constant temperature, the pressure varied between 1 bar and 10 bar, and for constant pressure, the temperature varied between 313.15 K and 423.15 K. For the rolled scenarios, specific investigations regarding accuracy were used, such as coefficient of determination (r^2^), *p* value and Pearson’s correlation (PCC). The achieved purposes were (i) the development of a predictive model that estimates the adsorption capacity of the chosen nanomaterials, varying with temperature and pressure, and (ii) the identification of each variable’s relative impact on the adsorption capacity.

The forecasted adsorption capacities of all the nanomaterials over NO (Supplementary Material—Figures S1 and S2) were determined with a high accuracy degree, supported by an r^2^ between 0.88% and 0.93% and a *p*_value_ < 0.05. The Pearson coefficient indicates a strong correlation between the predictors, P and T, and the response variable, a_NO._ PCC is situated (i) at the lower border of the specific domain, with a registered value of −0.97, for temperature, showing a high negative causal link, and (ii) at the upper border, 0.94 for pressure, demonstrating similar intensity causality, but in a positive direction.

With a *p*_value_ < 0.05 and an r^2^ between 0.81% and 0.90%, the predictions for the adsorption capacities of all the nanomaterials over SO_2_ are precise, emphasized in (Supplementary Material—Figures S3 and S4). The correlation matrix suggests similar causal links to the case of NO adsorption, with PCC values of 0.97 for the pressure variation scenario and −0.93 for the temperature variation scenario.

Detailed analysis of the data developed through projections maintained the temperature of 313.15 K as the generator of the highest values in terms of adsorption capacity. An increase in process pressure from 5 bar to 10 bar had a positive effect on adsorption capacity, but with insignificant variations of it. The doubling of pressure was not considered a viable solution in terms of profitability, bringing an increase of only 0.7–3% for NO and 3.1–5.9% for SO_2_, respectively.

## 4. Conclusions

In this study, the adsorption capacity of the developed nanomaterials (Fe-BTC, UiO-66, Cu-Zn-MCM-41) and commercial adsorbents (AC, ZMS-5 and SiO_2_) over NO and SO_2_ was investigated in a constant flow of test gas, Q 100 mL/min, a constant velocity of test gas, v 5 m/s and varying temperature, 313.15–373.15 K, and pressure, 1–5 bar, in a developed column reactor system.

With few exceptions, the adsorption processes for both NO and SO_2_ is materialized in correlation with pressure, respectively: (i) until breakthrough time, the temporal interval is longer in the case of tests performed at 1 bar compared to those performed at 5 bar, while (ii) the upper values of the adsorption capacity of the second and third intervals are in direct positive correlation with the pressure of 5 bar.

Notably, the third interval emerged as the most thermosensitive, with the increase in temperature causing the blocking of the active centers on the surface of the nanomaterial, which intervened in the adsorption mechanism and negatively affected performance.

The commercial catalysts selected for the study of NO adsorption recorded values similar to those described in the literature, arranged in ascending order of NO adsorption capacity (a_NO_) as follows: (i) AC, 16.00–20.70 cm^3^/g; (ii) ZSM-5, 13.79–16.80 cm^3^/g; and (iii) SiO_2_, 7.73–9.40 cm^3^/g. By comparison, the developed nanomaterials exhibited higher adsorption capacities. According to the recorded adsorption values, Fe-BTC had the highest affinity for NO, with 64.6–80.00 cm^3^/g, followed by UiO-66, with 48.73–57.33 cm^3^/g and Cu-Zn-MCM-41, with 46.00–48.00 cm^3^/g. This particular order of performance was maintained in the case of SO_2_ removal, with slightly smaller values for the adsorption capacity.

Demonstrating higher adsorption capacities than the commercial alternatives, the in-house developed nanomaterials present practical applicability in the energy sector, as part of the environmental hazard mitigation. The study concludes that both temperature and pressure significantly influence the adsorption capacity, underscoring the importance of these factors in optimizing adsorbtion processes.

## Figures and Tables

**Table 1:** The physical properties of the tested adsorbent materials under varying operating parameters for NO adsorption.

Adsorbent Materials	P bar	T K	m_material_ g	C_final_ NO ppmv	Interval I t_breakthrough_, min	Interval II min	Interval III min	a_NO_ cm^3^/g	ɳ_NO_ %	R_NO_ %
Fe-BTC	1	313.15	150	144.12	19	8	8	74.48	96.08	76.08
	1	373.15	150	144.30	17	10	8	64.60	96.20	75.35
	5	313.15	150	144.00	4	27	4	80.00	96.00	80.35
	5	373.15	150	145.00	4	26	5	66.67	96.67	69.00
UiO-66	1	313.15	150	146.00	19	10	6	50.67	97.33	77.66
	1	373.15	150	145.70	17	12	6	48.73	97.13	74.80
	5	313.15	150	145.70	4	18	13	57.33	97.13	52.17
	5	373.15	150	145.90	4	19	12	54.67	97.27	56.96
Cu-Zn-MCM-41	1	313.15	150	145.60	16	14	5	46.93	97.07	76.70
	1	373.15	150	145.40	15	15	5	46.00	96.93	77.46
	5	313.15	150	145.20	3	23	9	48.00	96.80	65.73
	5	373.15	150	145.25	3	21	11	47.50	96.83	57.53
AC	1	313.15	150	147.61	11	16	8	17.53	98.41	65.69
	1	373.15	150	148.00	12	14	9	16.00	98.67	65.16
	5	313.15	150	147.93	3	20	12	20.70	98.62	52.18
	5	373.15	150	148.12	3	18	14	18.80	98.75	48.76
SiO_2_	1	313.15	150	148.63	9	23	3	8.22	99.09	77.98
	1	373.15	150	148.84	10	19	6	7.73	99.23	68.12
	5	313.15	150	149.06	3	18	14	9.40	99.37	50.93
	5	373.15	150	149.12	3	18	14	8.80	99.41	50.82
ZSM-5	1	313.15	150	148.19	12	16	7	14.48	98.79	70.95
	1	373.15	150	148.12	11	17	7	13.79	98.75	70.93
	5	313.15	150	148.32	3	18	14	16.80	98.88	47.50
	5	373.15	150	148.39	3	18	14	16.10	98.93	49.98

**Table 2:** Elemental analysis of the tested adsorbent materials before and after the adsorption tests—nitrogen content.

Adsorbent Materials	Analyzed Parameter	Pressure/Temperature Variation			
		1 bar/313.15 K	1 bar/373.15 K	5 bar/313.15 K	5 bar/373.15 K
Fe-BTC	N_initial_ wt%	0.79	0.79	0.79	0.79
	N_final_ wt%	3.12	3.04	3.37	3.30
UiO-66	N_initial_ wt%	L_Q_ (<0.01)	L_Q_ (<0.01)	L_Q_ (<0.01)	L_Q_ (<0.01)
	N_final_ wt%	0.08	0.07	0.09	0.09
Cu-Zn-MCM-41	N_initial_ wt%	0.25	0.25	0.25	0.25
	N_final_ wt%	0.30	0.26	0.32	0.27
AC	N_initial_ wt%	0.51	0.51	0.51	0.51
	N_final_ wt%	0.66	0.60	0.78	0.71
SiO_2_	N_initial_ wt%	L_Q_ (<0.01)	L_Q_ (<0.01)	L_Q_ (<0.01)	L_Q_ (<0.01)
	N_final_ wt%	0.05	0.05	0.06	0.06
ZSM-5	N_initial_ wt%	L_Q_ (<0.01)	L_Q_ (<0.01)	L_Q_ (<0.01)	L_Q_ (<0.01)
	N_final_ wt%	0.09	0.08	0.11	0.10

Note. L_Q_—quantification detection limit of N.

**Table 3:** The physical properties of the tested adsorbent materials under varying operating parameters for SO_2_ adsorption.

Adsorbent Material	P bar	T K	m_material_ g	C_final_ SO_2_ ppmv	Interval I t_breakthrough_, min	Interval II min	Interval III min	a_SO2_ cm^3^/g	ɳ_SO2_ %	R_SO2_ %
Fe-BTC	1	313.15	150	69.80	15	6	14	52.00	93.07	60.33
	1	373.15	150	69.87	14	7	14	47.88	93.16	60.26
	5	313.15	150	70.27	4	17	14	63.07	93.69	50.93
	5	373.15	150	70.31	4	19	12	62.53	93.75	52.85
UiO-66	1	313.15	150	70.70	14	8	13	40.13	94.27	61.83
	1	373.15	150	70.71	13	10	12	37.18	94.28	62.49
	5	313.15	150	69.81	3	12	20	51.90	93.02	39.71
	5	373.15	150	70.09	3	17	15	49.10	93.45	52.28
Cu-Zn-MCM-41	1	313.15	150	69.51	10	10	15	36.60	92.68	55.04
	1	373.15	150	68.44	8	12	15	34.99	91.25	54.25
	5	313.15	150	70.67	3	15	17	43.30	94.23	45.82
	5	373.15	150	70.81	3	15	17	41.90	94.41	46.15
AC	1	313.15	150	72.71	10	13	12	15.27	96.95	60.22
	1	373.15	150	73.00	10	12	13	13.33	97.33	56.69
	5	313.15	150	73.73	4	14	17	16.93	98.31	49.29
	5	373.15	150	73.85	4	16	15	15.33	98.47	50.53
SiO_2_	1	313.15	150	73.25	9	12	14	10.50	99.81	53.72
	1	373.15	150	73.49	10	13	12	10.07	97.99	58.45
	5	313.15	150	73.78	3	16	16	12.20	98.37	45.92
	5	373.15	150	73.85	3	16	16	11.50	98.47	46.46
ZSM-5	1	313.15	150	73.15	10	14	11	12.33	97.53	65.09
	1	373.15	150	73.39	11	15	9	11.81	97.85	63.57
	5	313.15	150	73.51	3	16	16	14.90	98.01	48.99
	5	373.15	150	73.58	3	14	18	14.20	98.11	42.53

**Table 4:** Elemental analysis of the tested adsorbent materials before and after the adsorption tests—sulfur content.

Adsorbent Materials	Analyzed Parameter	Pressure/Temperature Variation			
		1 bar/313.15 K	1 bar/373.15 K	5 bar/313.15 K	5 bar/373.15 K
Fe-BTC	S_initial_ wt%	L_Q_ (<0.01)	L_Q_ (<0.01)	L_Q_ (<0.01)	L_Q_ (<0.01)
	S_final_ wt%	0.62	0.67	0.75	0.74
UiO-66	S_initial_ wt%	L_Q_ (<0.01)	L_Q_ (<0.01)	L_Q_ (<0.01)	L_Q_ (<0.01)
	S_final_ wt%	0.47	0.44	0.61	0.57
Cu-Zn-MCM-41	S_initial_ wt%	2.38	2.38	2.38	2.38
	S_final_ wt%	2.80	2.92	3.25	3.22
AC	S_initial_ wt%	1.34	1.34	1.34	1.34
	S_final_ wt%	1.76	1.40	2.07	2.16
SiO_2_	S_initial_ wt%	0.80	0.80	0.80	0.80
	S_final_ wt%	0.95	0.90	1.10	1.00
ZSM-5	S_initial_ wt%	L_Q_ (<0.01)	L_Q_ (<0.01)	L_Q_ (<0.01)	L_Q_ (<0.01)
	S_final_ wt%	0.29	0.28	0.35	0.33

Note. L_Q_—quantification detection limit of S.

## Data Availability

Data are contained within the article.

## Supplementary Materials

The following supporting information can be downloaded at: https://www.mdpi.com/article/10.3390/nano14100816/s1. Figure S1: Synthesized nanomaterials adsorption capacity forecasts for NO; Figure S2: Commercial materials adsorption capacity forecasts for NO; Figure S3: Synthesized nanomaterials adsorption capacity forecasts for SO_2_; Figure S4: Commercial materials adsorption capacity forecasts for SO_2_.
