# Supplementary material for: A Study on the Ability of Nanomaterials to Adsorb NO and SO2 from Combustion Gases and the Effectiveness of Their Separation
Source: Nanomaterials (Basel). 2024 May 7;14(10):816. doi: 10.3390/nano14100816 (PMC11123805; doi:10.3390/nano14100816)
Supplement: Supplementary file 1 [file nanomaterials-14-00816-s001.zip › nanomaterials-2982082-supplementary.pdf]

- Supplementary materials -

A Study on the Ability of Nanomaterials to Adsorb NO and SO_2 from Combustion Gases and the Effectiveness of Their Separation

Marius Constantinescu [1], Felicia Bucura [1,*], Antoaneta Roman [1,*], Oana Romina Botoran [1], Roxana-Elena Ionete [1], Stefan Ionut Spiridon [1], Eusebiu Ilarian Ionete [1], Anca Maria Zaharioiu [1], Florian Marin [1,2], Silviu-Laurentiu Badea [1] and Violeta-Carolina Niculescu [1,*]

1 National Research and Development Institute for Cryogenic and Isotopic Technologies—ICSI Ramnicu Valcea, 4th Uzinei Street, 240050 Ramnicu Valcea, Romania; marius.constantinescu@icsi.ro (M.C.)

2 Faculty of Agricultural Sciences, Food Industry and Environmental Protection, "Lucian Blaga" University of Sibiu, 7-9 I. Ratiu Str., 550012 Sibiu, Romania

* Correspondence: felicia.bucura@icsi.ro (F.B.); antoaneta.roman@icsi.ro (A.R.); violeta.niculescu@icsi.ro (V.-C.N.)

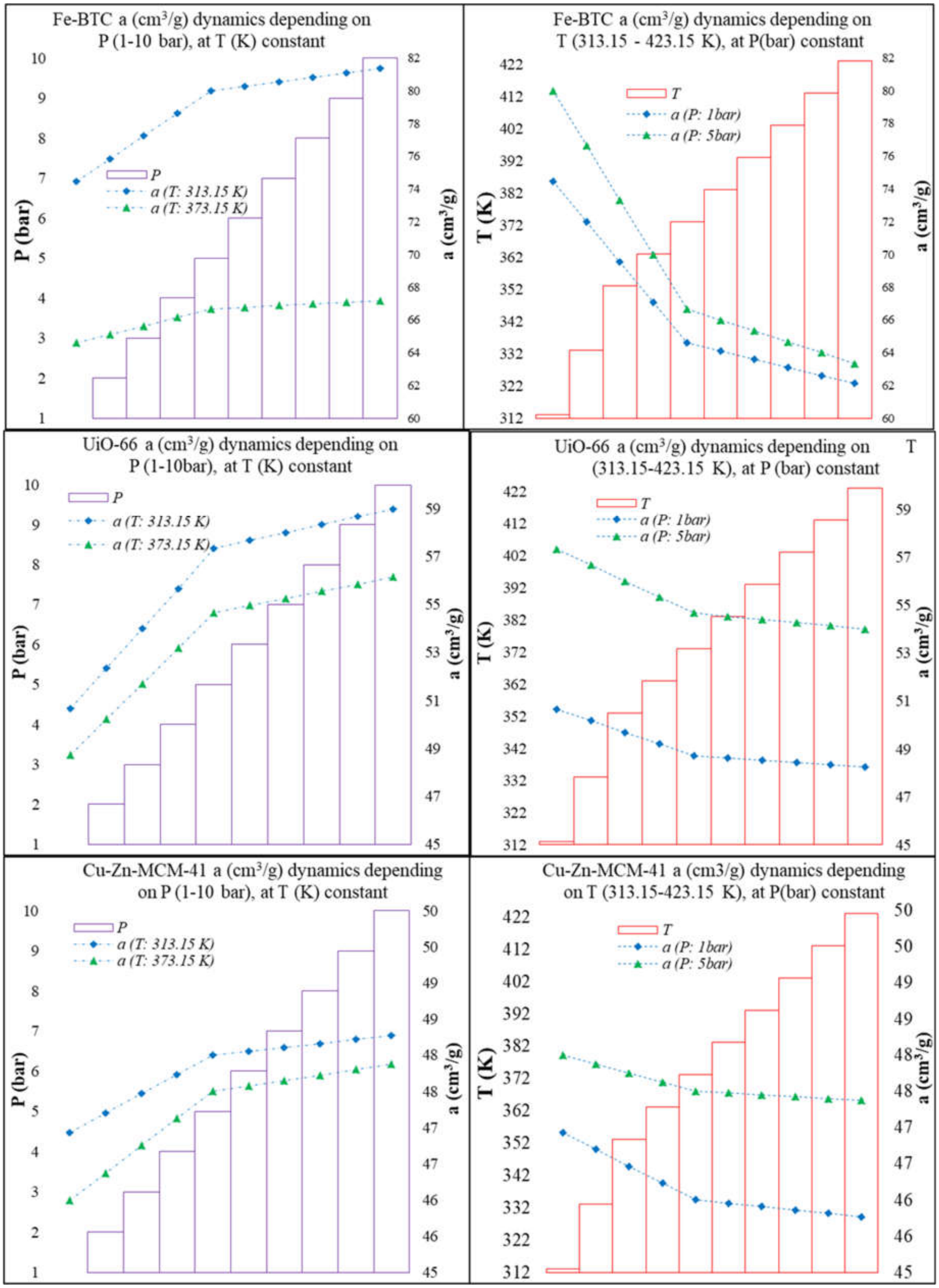

Figure S1. Synthesized nanomaterials adsorption capacity forecasts for NO.

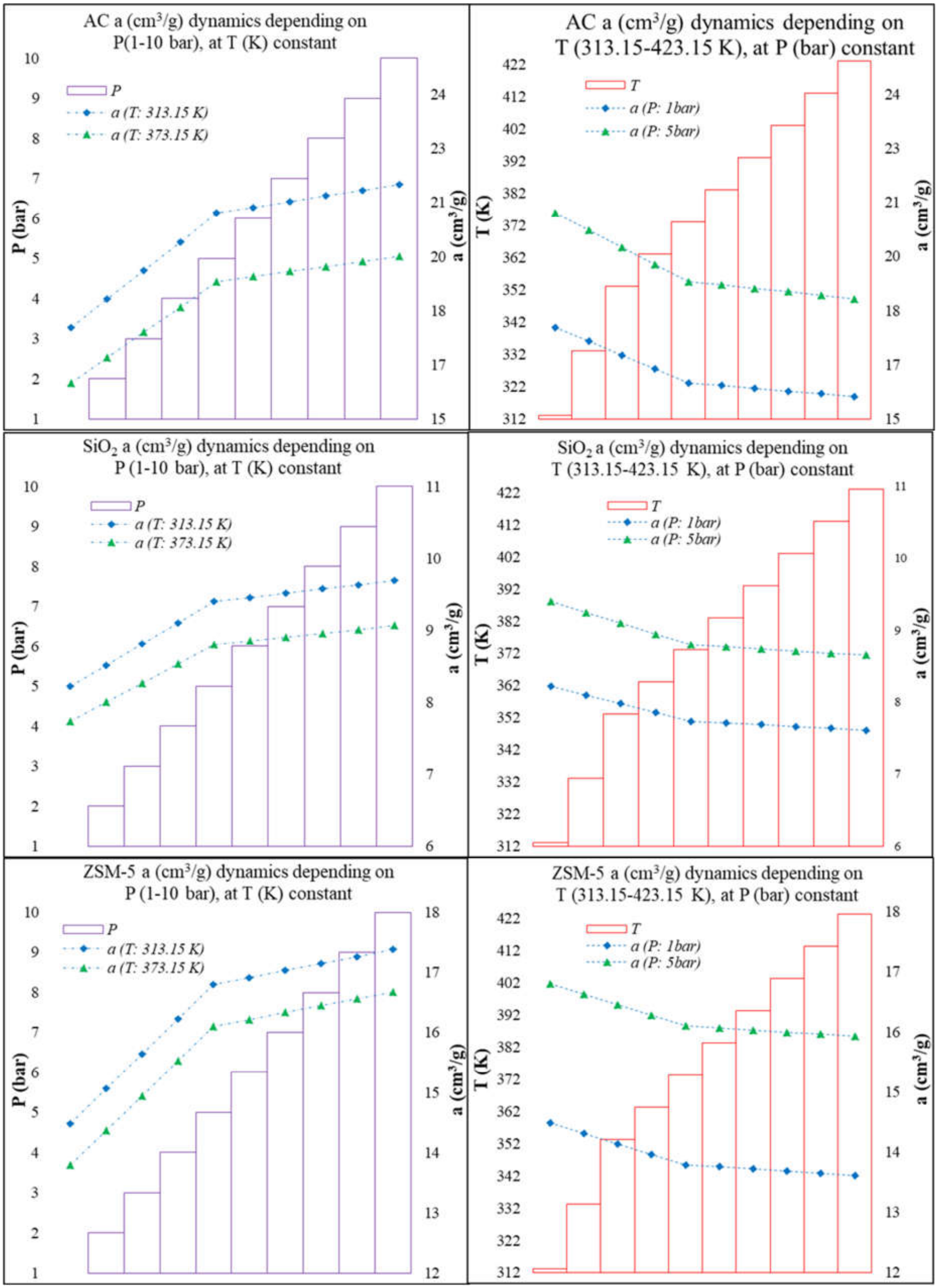

Figure S2. Commercial materials adsorption capacity forecasts for NO.

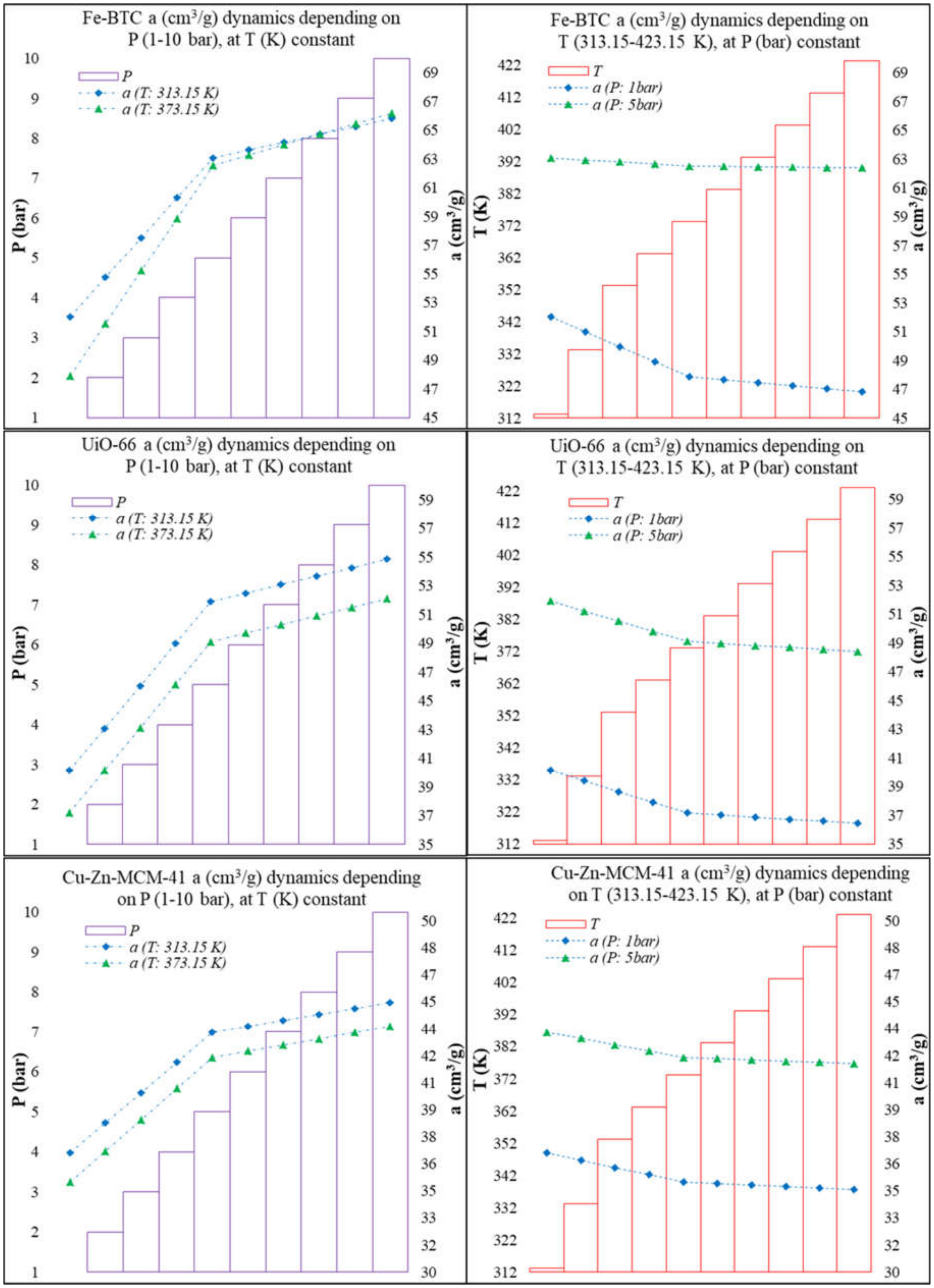

Figure S3. Synthesized nanomaterials adsorption capacity forecasts for SO_2.

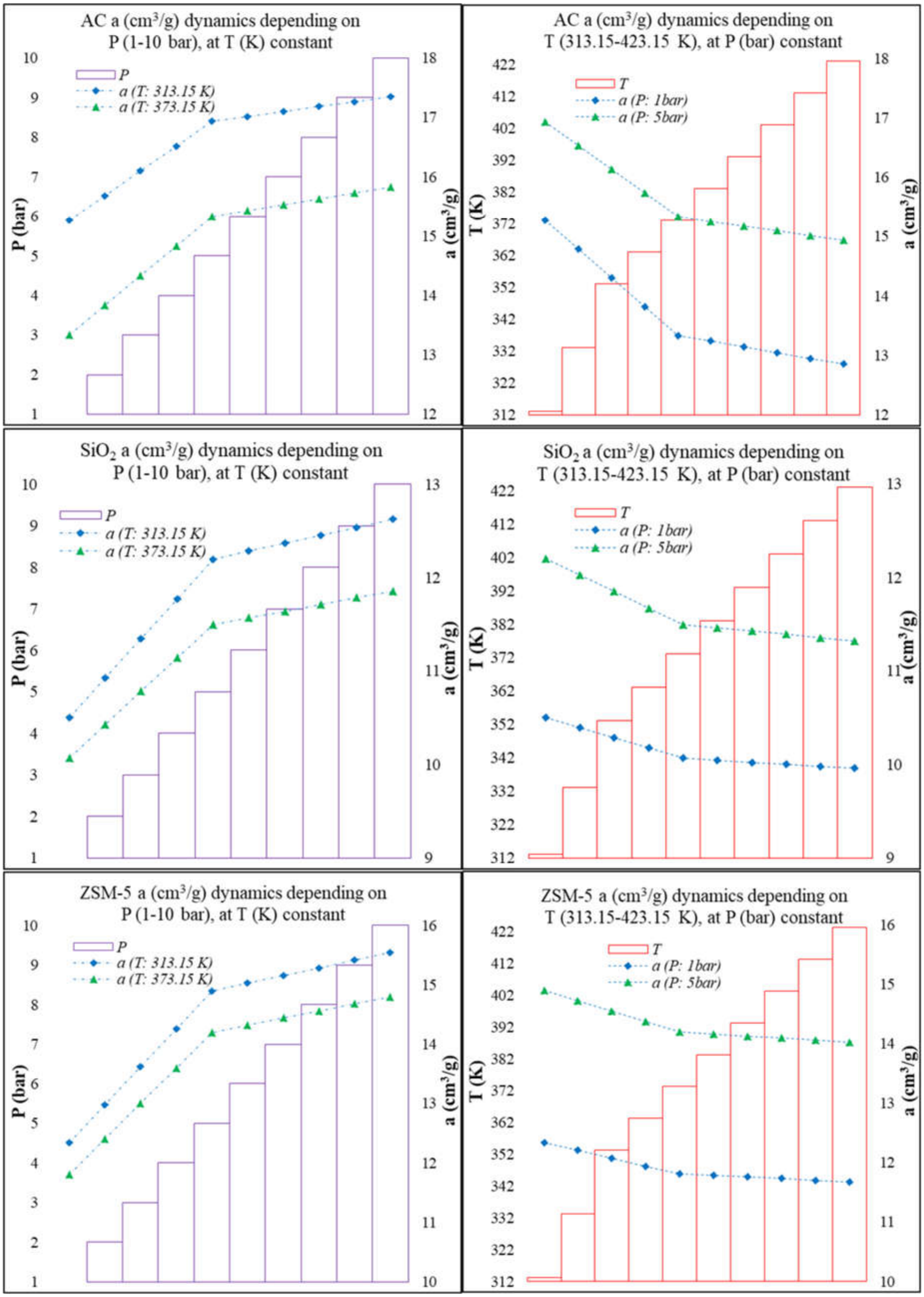

Figure S4. Commercial materials adsorption capacity forecasts for SO_2.
